# A Man With Chest Pain After An Assault – A Case Report

**DOI:** 10.21980/J8J93S

**Published:** 2024-07-31

**Authors:** Mi Song Kim, Francis Gan, Karl Nimtz, Daniel Ng, John Costumbrado

**Affiliations:** * Riverside Community Hospital, Department of Emergency Medicine, Riverside, CA; ^ University of California, Riverside, School of Medicine, Riverside, CA

## Abstract

This case report highlights an uncommon sequelae of chest wall trauma that should be evaluated for patients presenting with similar history and symptoms. A 60-year-old man presented to the emergency department (ED) with swelling, fever, and chest wall pain two days after an assault with blunt chest wall trauma. On exam, there was a suspected chest wall abscess, verified on computed tomography (CT) with associated displaced midsternal fracture. This patient was admitted for abscess incision and drainage. While uncommon, chest wall abscess formation is an important condition that should be considered as a differential diagnosis in any patient presenting with chest wall pain post blunt trauma. With few reported similar presentations in the literature, this case is an important addition in a likely underreported phenomenon that requires prompt evaluation and treatment.

**Topics:**

Blunt chest trauma, chest wall abscess, sternal fracture complication.

[Fig f1-9-3-v1] [Fig f2-9-3-v1] [Fig f3-9-3-v1]

**Figure f1-9-3-v1:**
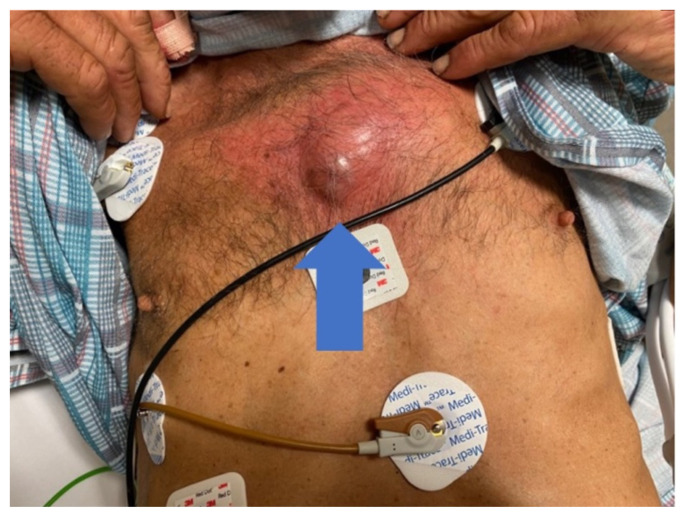


**Figure f2-9-3-v1:**
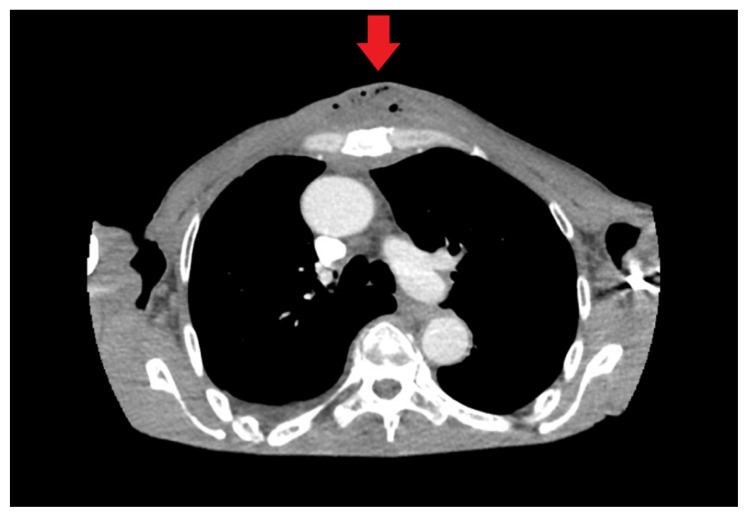


**Figure f3-9-3-v1:**
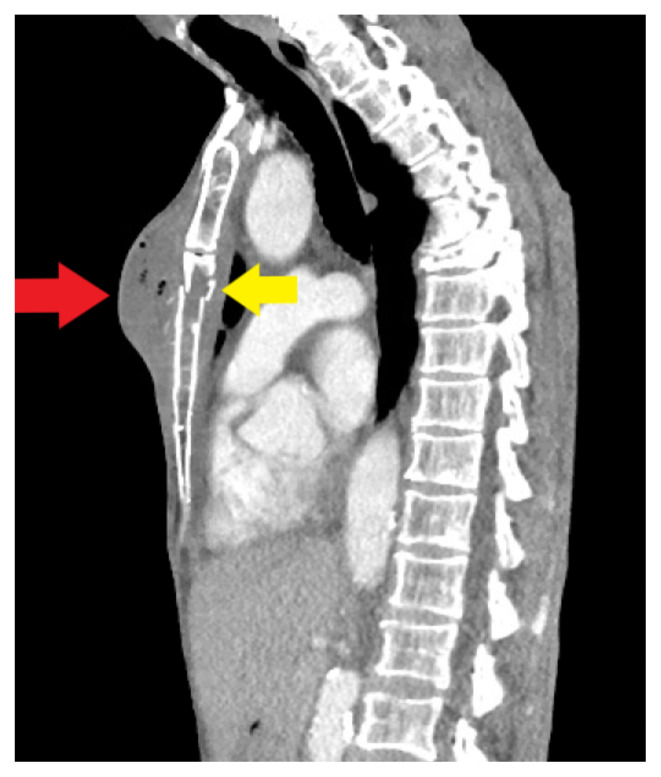


## Brief introduction

Chest wall abscesses are a rare clinical phenomenon that arise secondary to tissue infection, osteomyelitis of the ribs, infection of the costochondral junction or sternoclavicular joint, or blunt chest trauma.^1^ Blunt chest trauma is a rare factor leading to chest wall abscess formation, seeing that very few case reports are currently published regarding this phenomenon. This case report reviews chest wall abscess as a complication of sternal fracture in the setting of a blunt chest trauma, including the different ways this phenomenon can be diagnosed and managed. Written consent was obtained for publishing the images as a case report.

## Presenting concerns and clinical findings

A 60-year-old undomiciled male with a history of hypertension, tobacco use, and illicit fentanyl use presented to the ED with worsening midsternal chest pain and chest wall swelling status post an assault two days prior. He did not seek medical attention initially when the injury occurred. The patient reported someone stepped on his chest and denied any other symptoms. He had no significant family history. Physical examination revealed an 8-cm area of induration, erythema, and fluctuance in the mid-sternal chest wall (blue arrow) with chest wall tenderness but no crepitus or instability. His blood pressure was 167/102 mm Hg, and his other vitals were within normal limits. The rest of his exam was unremarkable.

## Significant findings

On exam, we found a suspected chest wall abscess with surrounding erythema (blue arrow). The patient underwent CT of the chest which showed a comminuted displaced midsternal fracture (yellow arrow) with moderate fluid and air anteriorly (red arrow), consistent with an abscess. His laboratory results had no significant abnormalities.

## Patient course

We consulted trauma surgery for further evaluation and management of the patient’s traumatic injuries. He was admitted for infection and pain control. The trauma surgery team performed an incision and drainage of the abscess and sent the fluid for cultures. The patient tolerated the procedure well without complications. He was started on vancomycin due to his risk factors for methicillin-resistant *Staphylococcus aureus* (MRSA), then was transitioned to oral sulfamethoxazole-trimethoprim. He was clinically improving and had no significant abnormalities in his subsequent laboratory results. He was discharged on admission day three with sulfamethoxazole-trimethoprim for seven days, wound care instructions and supplies, and plans for outpatient follow-up. Unfortunately, no further documentation was available in our electronic medical records regarding follow-up visits after discharge, and we were unsuccessful in contacting the patient to discuss patient-reported outcomes.

## Discussion

In previous case reports of blunt chest trauma, abscesses were detected five days to two months after the initial trauma with or without the presence of a sternal wall fracture.^2^–^11^ Trauma, in general, is a risk factor for abscess formation in the body, along with immunosuppression and impaired circulation.^12^ Based on these case reports, no specific type of blunt chest trauma has been associated with increased risk of abscess formation.^2^–^11^ Though we do not believe that the abscess could have been prevented if patient presented at the time of injury, it would be interesting to see studies regarding whether certain patient risk factors or type of blunt trauma could warrant prophylactic antibiotic treatment for prevention of infection and abscess formation. Staphylococcus aureus is the most common causative organism.^2^–^8^

These abscesses are diagnosed by ultrasound (US) or CT.^1^–^9^ Ultrasound of any body part is able to detect abscesses with a sensitivity of 95–97% and specificity of 83–85%, while CT had a sensitivity of 77% and specificity of 91%.^13^–^14^ Though sensitivity and specificity of CT may be lower than US, CT has high sensitivity, with up to 100% in one study; therefore, CT may be more beneficial for evaluating chest wall infection or abscess in the setting of a sternal fracture.^15^–^16^

Treatment for chest wall abscesses varies depending on the severity of infection, including systemic antibiotics, bedside incision and drainage, and surgical debridement or chest wall reconstruction.^2^–^11^,^17^ In most of the reviewed case reports, patients recovered well with some cases having no recurrence in six months.^1^,^8^ Though we did not find specific case reports regarding complications from chest wall abscesses, fulminant infections can occur with delayed treatment especially in aged or immunocompromised patients; thus, prompt and appropriate management is important in treating chest wall abscesses.^1^

In conclusion, physicians should consider infectious etiologies, including cellulitis and abscess, in patients presenting with worsening chest pain following blunt chest trauma. A thorough physical exam and, if needed, diagnostic imaging with US or a CT scan of chest will allow a proper diagnosis of chest wall abscess. Initial treatment includes incision and drainage of the abscess in addition to systemic antibiotics covering for MRSA, with good prognosis if detected and treated in a timely manner.

## Supplementary Information












