# Supplementary figures and images for: A Man With Chest Pain After An Assault – A Case Report
Source: J Educ Teach Emerg Med. 2024 Jul 31;9(3):V1–4. doi: 10.21980/J8J93S (PMC11312881; doi:10.21980/J8J93S)

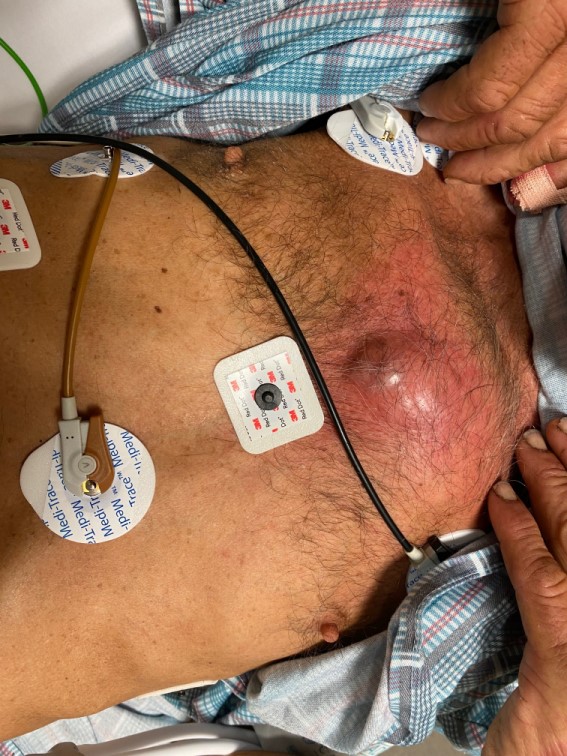

Supplement: Supplementary file 1 [file 9-3-V1-Supp1.jpg]

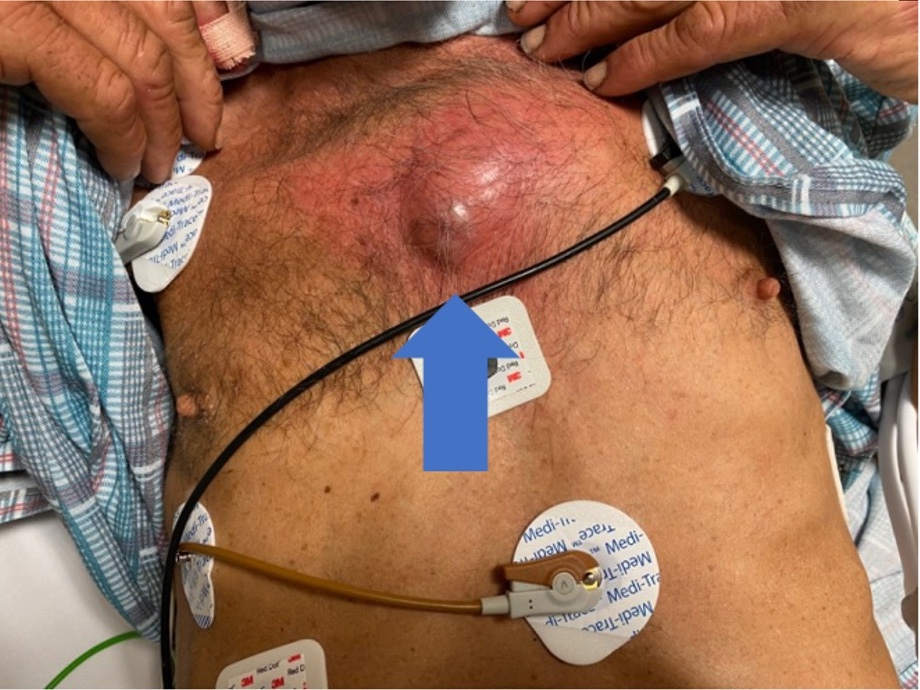

Supplement: Supplementary file 2 [file 9-3-V1-Supp2.jpg]

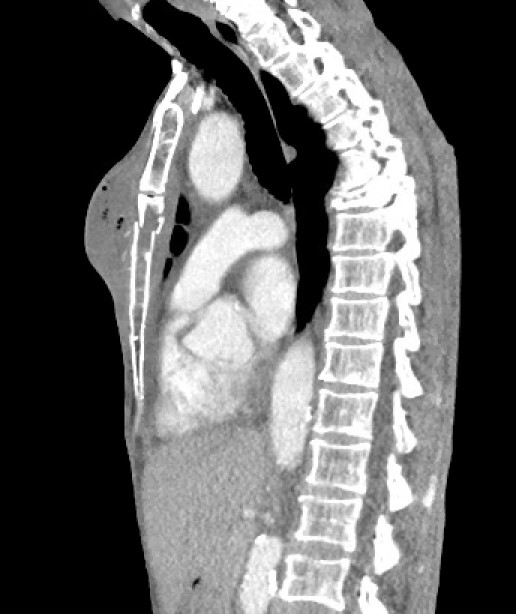

Supplement: Supplementary file 3 [file 9-3-V1-Supp3.jpg]

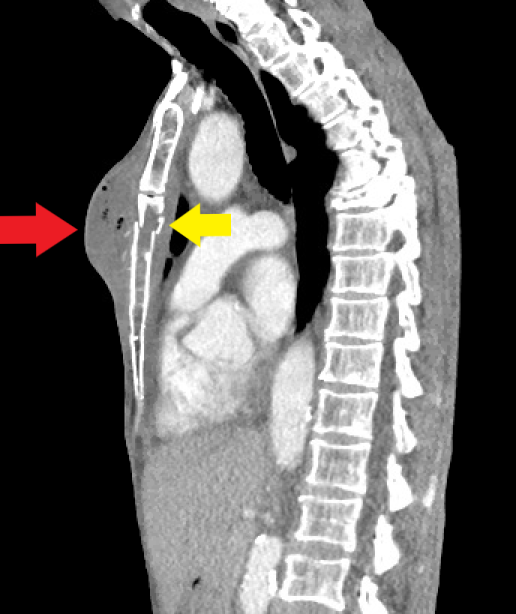

Supplement: Supplementary file 4 [file 9-3-V1-Supp4.png]

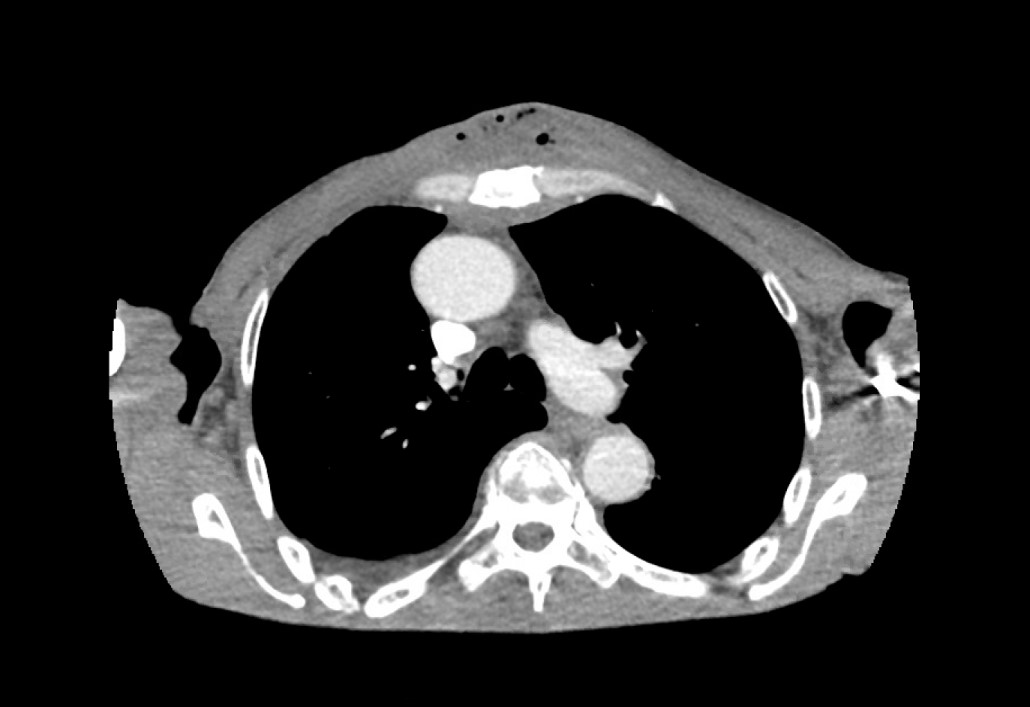

Supplement: Supplementary file 5 [file 9-3-V1-Supp5.jpg]

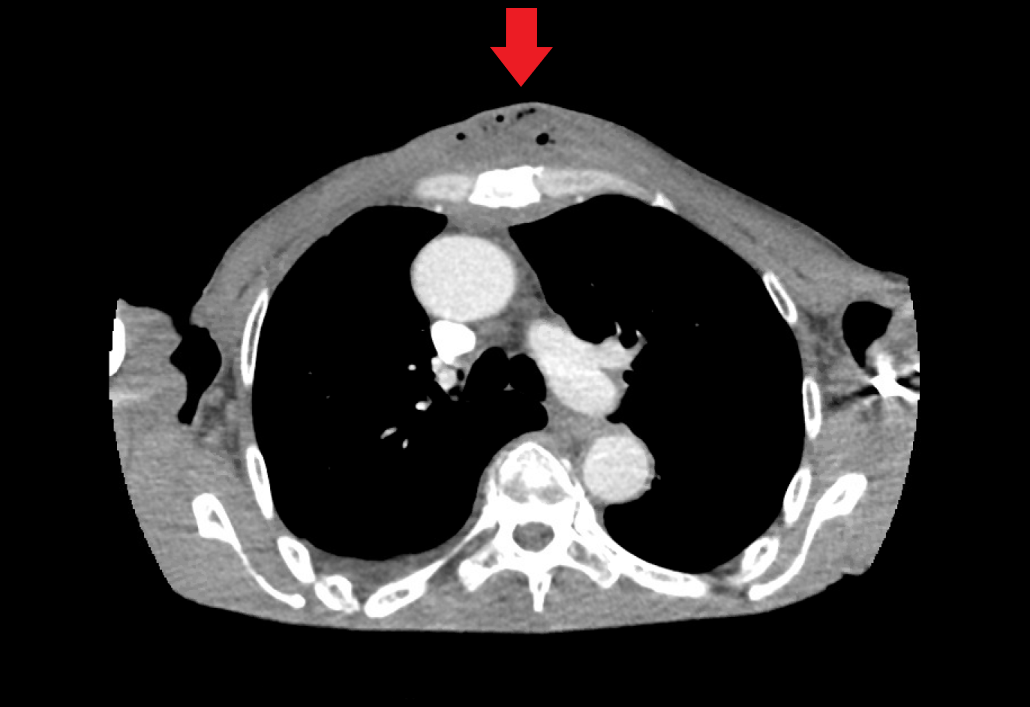

Supplement: Supplementary file 6 [file 9-3-V1-Supp6.png]
